# Effect of a cash transfer intervention on memory decline and dementia probability in older adults in rural South Africa

**DOI:** 10.1073/pnas.2321078121

**Published:** 2024-09-19

**Authors:** Molly Rosenberg, Erika T. Beidelman, Xiwei Chen, Chodziwadziwa Whiteson Kabudula, Audrey Pettifor, Darina T. Bassil, Lisa Berkman, Kathleen Kahn, Stephen Tollman, Lindsay C. Kobayashi

**Affiliations:** ^a^Department of Epidemiology and Biostatistics, Indiana University School of Public Health-Bloomington, Bloomington, IN 47405; ^b^South African Medical Research Council/Wits Rural Public Health and Health Transitions Research Unit (Agincourt), School of Public Health, Faculty of Health Sciences, University of the Witwatersrand, Johannesburg 2193, South Africa; ^c^Department of Epidemiology and Biostatistics, Biostatistics Consulting Center, Indiana University School of Public Health-Bloomington, Bloomington, IN 47405; ^d^Department of Epidemiology, University of North Carolina-Chapel Hill Gillings School of Global Public Health, Chapel Hill, NC 27599; ^e^Center for Population and Development Studies, Harvard TH Chan School of Public Health, Cambridge, MA 02138; ^f^Department of Epidemiology and Global Health, Umeå University, Umeå 901 85, Sweden; ^g^Department of Epidemiology, Center for Social Epidemiology and Population Health, University of Michigan School of Public Health, Ann Arbor, MI 48109

**Keywords:** cash transfer, dementia, memory decline, South Africa

## Abstract

Evidence on cash transfers as a population-level intervention to support healthy cognitive aging in low-income settings is sparse. We assessed the effect of a cash transfer intervention on cognitive aging outcomes in older South African adults. We leveraged the overlap in the sampling frames of a Phase 3 randomized cash transfer trial [HIV Prevention Trial Network (HPTN) 068, 2011–2015] and an aging cohort [Health and Aging in Africa: A Longitudinal Study of an INDEPTH Community (HAALSI), 2014–2022] in rural Mpumalanga Province, South Africa. In 2011/12, young women and their primary caregivers were randomly assigned 1:1 to receive a monthly cash transfer or control. In 2014/2015, 862 adults aged 40+ y living in trial households were enrolled in the HAALSI cohort, with cognitive data collected in three waves over 7 y. We estimated the impact of the intervention on rate of memory decline and dementia probability scores. Memory decline in the cash transfer arm was 0.03 SD units (95% CI: 0.002, 0.05) slower per year than in the control arm. Dementia probability scores were three percentage points lower in the cash transfer arm than the control arm (β = −0.03; 95% CI: −0.05, −0.001). Effects were consistent across subgroups. A modestly sized household cash transfer delivered over a short period in mid- to later-life led to a meaningful slowing of memory decline and reduction in dementia probability 7 y later. Cash transfer programs could help stem the tide of new dementia cases in economically vulnerable populations in the coming decades.

## Significance

As the global population ages, the burden of Alzheimer’s Disease and Related Dementias (ADRD) is growing rapidly. ADRDs are the result of etiological processes that are shaped, in part, by financial resources accumulated across the life course; thus, programs that increase income through cash transfers could support healthy cognitive aging. Our study in rural South African older adults evaluates the impact of a randomized cash transfer intervention on long-term and rigorously measured cognitive outcomes over 7 y of follow-up. We found that a relatively modest household cash transfer intervention delivered over 3 y led to meaningfully slower memory decline and lower dementia probability.

As the global population ages, the burden of Alzheimer’s Disease and Related Dementias (ADRD) is growing rapidly. By 2050, nearly 75% of the projected 153 million global ADRD cases will be in low- and middle-income countries (LMICs) (1, 2). ADRDs cause significant morbidity and mortality, accounting for over 33 million disability-adjusted life years lost in 2019 and expected to triple by 2050 (3). ADRD also has high economic costs. By 2050, the global economic burden of ADRD is projected to reach nearly $17 trillion, with LMICs accounting for 65% of this total (4). Reducing economic vulnerabilities in older adults could play a critical role in preventing or delaying ADRDs (5), as they are the result of etiological processes that are shaped, in part, by financial resources accumulated across the life course (6). Translating this knowledge into successful interventions in low-income settings will be key to preventing ADRD cases in LMICs and averting the worst of the projected consequences for the global aging community.

Programs that increase income through direct cash payments to economically vulnerable individuals or households (“cash transfers”), show promise as an intervention to support healthy cognitive aging. The potential use of cash transfers for ADRD protection is grounded in the theory of cognitive reserve. Cognitive reserve is thought to confer resilience of day-to-day cognitive function to aging-related brain pathology or injury (7). It is theorized to build up over the life course through accumulated experience with cognitively stimulating exposures, such as sources of learning, social engagement, and leisure activities. Cash transfers could plausibly support investments that provide cognitive stimulation, such as leisure activities (e.g. access to media through television or the Internet) or enabling social connections (e.g. telephones, transportation) (8). Financial resources from cash transfers could also be useful for ADRD prevention in improving nutrition, providing healthier lifestyles for chronic disease prevention, and accessing healthcare for chronic disease management (9). Cash transfer programs have expanded widely across LMICs in the last two decades (10), with many more countries looking to expand existing or pilot new programs. Rigorous evidence is now needed to support LMIC decision-making around whether and how to invest in cash transfer programs to promote cognitive health in their aging populations.

Despite their promise, evidence on the relationship between cash transfers and ADRD is sparse. Pensions can be classified as cash transfers targeted to those in retirement age, and have shown positive associations with aging-related health outcomes such as reduced mortality and maintained functional status in South Africa (11, 12). Evidence on cognitive aging outcomes is less common. Prior studies demonstrated a positive relationship between pension income and cognitive function in the United States (13) and South Africa (14), with longitudinal cognitive function in South Korea (15), and with memory decline in China and Mexico (16, 17). However, randomized cash transfer experiments are rare, which limits causal inference. Other key research gaps include the need for more rigorous longitudinal follow-up for ADRD outcome measures, and a need for expanded representation of populations in sub-Saharan Africa, where much of the future ADRD burden is projected to occur.

In this study, we leveraged the household overlap of participants in a randomized cash transfer trial [HIV Prevention Trial Network (HPTN) 068, 2011 to 2015] and an aging cohort study [“Health and Aging in Africa: A Longitudinal Study of an INDEPTH Community” (HAALSI), 2014 to 2022] in a rural South African community. We estimated the causal effect of the cash transfer intervention on longitudinal memory decline and dementia probability in older adults aged at least 40 y across three waves of cognitive data collection over 7 y of follow-up. We further explored whether effects differed by key sociodemographic variables and by duration and nature of cash transfer receipt.

## Results

**Fig. 1.**
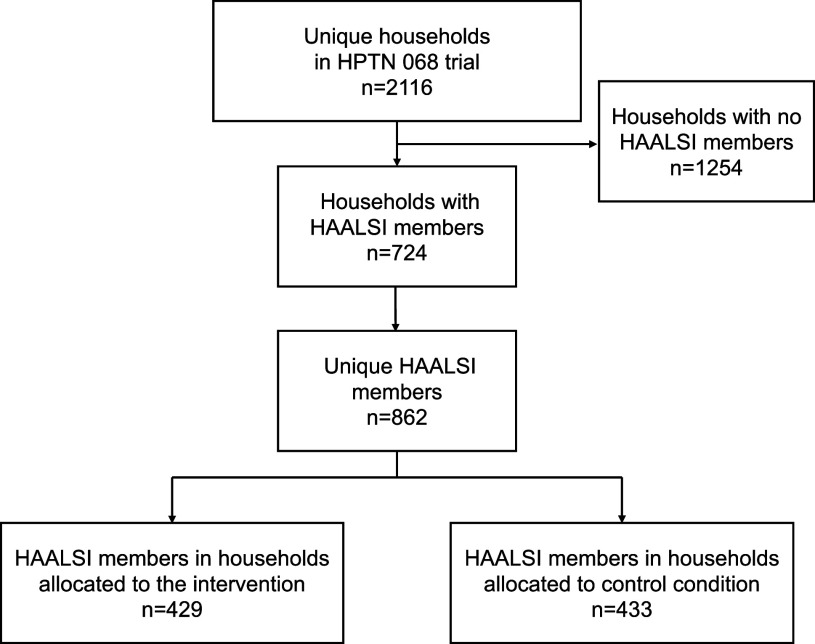
Flow diagram to arrive at this study’s analytic sample.

Overall, n = 862 HAALSI participants lived in HPTN 068 trial households (n = 429 intervention, n = 433 control; Fig. 1 and Table 1). The mean age of the sample at HAALSI baseline was 61 y (SD 12.1), with just over half female (56%), about a third former Mozambican refugees (32%), and about two-thirds married/cohabitating with a partner (63%). Reflective of the limited educational opportunities in the area, nearly half (44%) of the sample reported no formal education. Most of the sample was related to the index young woman as parents (27% father, 27% mother) or grandparents (8% grandfather, 16% grandmother), and 43% were their primary caregivers. Most households included a young adult aged 18 to 25 y (95%), and most included a child who was age-eligible for the Child Support Grant (CSG) (71%). Nearly half (48%) of the older adults were themselves age-eligible for the Older Persons’ Grant (OPG). Intervention arm participants received the cash transfer for differing durations, depending on the school grade of the index young woman: <1 y (24%), 1 to 2 y (34%), and 2+ y (43%). As expected from randomization, there were no meaningful differences between the study arms in the distribution of any of these sociodemographic variables.

**Table 1.** Sociodemographic characteristics of n = 862 adult HAALSI household members of HPTN 068 trial participants at HAALSI baseline in 2014/2015, by cash transfer intervention arm

	Total (n = 862) Mean (SD)	Cash transfer (n = 429) Mean (SD)	No cash transfer (n = 433) Mean (SD)	*P*-value^*^
Age (years)				
HPTN 068 baseline	58.3 (11.5)	58.3 (10.9)	58.4 (12.1)	0.793
HAALSI baseline	61.0 (12.1)	60.9 (11.5)	61.0 (12.6)	0.839
Household size	7.20 (3.88)	7.39 (3.89)	7.02 (3.87)	0.183
Memory scores (W3)	0.387 (0.699)	0.414 (0.722)	0.362 (0.676)	0.355
Dementia probability (W3)	0.103 (0.107)	0.096 (0.094)	0.111 (0.119)	0.176
	Freq. (%)	Freq. (%)	Freq. (%)	*P*-value^†^
Sex				0.503
Male	377 (43.7)	193 (45.0)	184 (42.5)	
Female	485 (56.3)	236 (55.0)	249 (57.5)	
Born in South Africa				0.224
Yes	581 (67.6)	298 (69.6)	283 (65.5)	
No	279 (32.4)	130 (30.4)	149 (34.5)	
Missing	2	1	1	
Marital status				0.560
Divorced/separated	58 (6.7)	24 (5.6)	34 (7.9)	
Married/cohabiting	545 (63.2)	273 (63.6)	272 (62.8)	
Single	28 (3.2)	13 (3.0)	15 (3.5)	
Widowed	231 (26.8)	119 (27.7)	112 (25.9)	
Wealth index				0.237
Quintile 1	132 (15.3)	66 (15.4)	66 (15.2)	
Quintile 2	166 (19.3)	84 (19.6)	82 (18.9)	
Quintile 3	197 (22.9)	92 (21.4)	105 (24.2)	
Quintile 4	190 (22.0)	107 (24.9)	83 (19.2)	
Quintile 5	177 (20.5)	80 (18.6)	97 (22.4)	
Years of education				0.275
No education	378 (44.0)	179 (41.7)	199 (46.3)	
<12 y	423 (49.2)	223 (52.0)	200 (46.5)	
≥12 y	58 (6.8)	27 (6.3)	31 (7.2)	
Missing	3	0	3	
Attrition by Wave 3				0.690
Yes	195 (22.6)	100 (23.3)	95 (21.9)	
No	667 (77.4)	329 (76.7)	338 (78.1)	
Relationship to young woman				0.425
Father	229 (26.6)	117 (27.3)	112 (25.9)	
Mother	231 (26.8)	106 (24.7)	125 (28.9)	
Grandfather	66 (7.7)	39 (9.1)	27 (6.2)	
Grandmother	134 (15.5)	66 (15.4)	68 (15.7)	
Other	202 (23.4)	101 (23.5)	101 (23.3)	
Primary caregiver^‡^				0.363
Yes	341 (42.8)	163 (41.1)	178 (44.5)	
No	456 (57.2)	234 (58.9)	222 (55.5)	
Missing	65	32	33	
Young adult (18 to 25 y) in household				0.118
Yes	817 (94.8)	401 (93.5)	416 (96.1)	
No	45 (5.2)	28 (6.5)	17 (3.9)	
Household CSG eligibility				0.644
Yes	614 (71.2)	302 (70.4)	312 (72.1)	
No	248 (28.8)	127 (29.6)	121 (27.9)	
Individual OPG eligibility				0.834
Yes	416 (48.3)	205 (47.8)	211 (48.7)	
No	446 (51.7)	224 (52.2)	222 (51.3)	
Cash transfer duration				-
<1 y	-	89 (23.7)	-	
1 to 2 y	-	127 (33.8)	-	
2+ y	-	160 (42.6)	-	
Missing	-	53	-	

^*^Wilcoxon rank sum test.

^†^Pearson's chi-squared test.

^‡^Primary caregiver status assessed for participants who were parents or grandparents of the HPTN 068 young women only. Participants with other relationships were coded as missing if a primary caregiver was not otherwise noted for the young woman.

### Memory Decline.

Memory decline was slower for those who received the cash transfer intervention relative to those who did not (Table 2 and *SI Appendix*, Fig. S1). As expected, in the full analytic sample, the time coefficient was negative (β = −0.03 SD units; 95% CI: −0.06, −0.003), indicating the average decline in memory score per year of follow-up in the trial control arm. Memory decline in the cash transfer arm was 0.03 SD units (95% CI: 0.002, 0.05) slower per year compared to the control arm, essentially counteracting the magnitude of the aging effect. A model that allowed for nonlinear changes in memory function over time produced results that were consistent with our main findings, indicating that those in the cash transfer arm began to outperform the control arm in memory function at Wave 2, and that this difference was maintained at Wave 3 (*SI Appendix*, Fig. S2 and Table S1).

**Table 2.** Impact of the cash transfer intervention on memory slopes, with coefficients from a generalized linear mixed model,^*^ n = 862 older adults age 40 y and older, 2014 to 2022

		Intercept			Cash transfer			Time (years)			Cash transfer*Time			
	N	ß	95% CI	*P*-value	ß	95% CI	*P*-value	ß	95% CI	*P*-value	ß	95% CI	*P*-value	*P*-value for interaction
Overall	862	0.48	(0.32, 0.64)	<0.001	−0.04	(−0.18, 0.09)	0.519	−0.03	(−0.06, −0.003)	0.032	0.03	(0.002, 0.05)	0.036	-
Stratified by sex														0.427
Female	485	0.43	(0.23, 0.63)	<0.001	−0.05	(−0.21, 0.12)	0.586	−0.02	(−0.06, 0.02)	0.290	0.02	(−0.01, 0.05)	0.240	
Male	377	0.57	(0.30, 0.83)	<0.001	−0.07	(−0.28, 0.15)	0.553	−0.05	(−0.10, −0.004)	0.034	0.04	(−0.001, 0.08)	0.055	
Stratified by age at HAALSI baseline														0.586
<50 y old	145	0.59	(0.23, 0.95)	0.002	0.10	(−0.20, 0.40)	0.533	0.03	(−0.04, 0.10)	0.446	0.03	(−0.03, 0.09)	0.328	
50 to 69 y old	515	0.70	(0.50, 0.90)	<0.001	−0.01	(−0.18, 0.16)	0.873	−0.06	(−0.10, −0.02)	0.002	0.01	(−0.02, 0.04)	0.415	
70+ y old	202	−0.12	(−0.47, 0.24)	0.519	−0.14	(−0.40, 0.12)	0.292	−0.02	(−0.09, 0.05)	0.553	0.05	(−0.003, 0.10)	0.064	
Stratified by age at HPTN 068 baseline														0.498
<60 y old	481	0.84	(0.63, 1.04)	<0.001	−0.09	(−0.26, 0.08)	0.306	−0.06	(−0.09, −0.02)	0.005	0.02	(−0.01, 0.05)	0.238	
60+ y old	336	0.005	(−0.26, 0.27)	0.971	−0.09	(−0.29, 0.11)	0.392	−0.02	(−0.07, 0.03)	0.517	0.04	(0.001, 0.08)	0.047	
Stratified by household size														0.978
Above median (6)	421	0.49	(0.26, 0.71)	<0.001	0.03	(−0.15, 0.22)	0.717	−0.04	(−0.08, 0.002)	0.060	0.03	(−0.01, 0.06)	0.147	
At or below median (6)	441	0.47	(0.24, 0.71)	<0.001	−0.11	(−0.31, 0.09)	0.272	−0.03	(−0.07, 0.02)	0.234	0.03	(−0.01, 0.06)	0.133	
Stratified by young adult (18 to 25 y) household membership														0.896
Yes	817	0.53	(0.36, 0.70)	<0.001	−0.04	(−0.18, 0.09)	0.531	−0.04	(−0.07, −0.01)	0.010	0.03	(0.002, 0.05)	0.035	
No	45	−0.19	(−0.92, 0.55)	0.616	−0.01	(−0.71, 0.68)	0.972	0.06	(−0.09, 0.21)	0.447	0.02	(−0.11, 0.16)	0.746	
Stratified by SES														0.895
Above median	422	0.66	(0.43, 0.88)	<0.001	−0.04	(−0.23, 0.16)	0.704	−0.07	(−0.11, −0.02)	0.002	0.03	(−0.01, 0.06)	0.112	
Below median	439	0.32	(0.09, 0.55)	0.006	−0.06	(−0.25, 0.13)	0.561	−0.004	(−0.05, 0.04)	0.858	0.03	(−0.01, 0.06)	0.138	
Stratified by education														0.668
≥12 y	58	1.30	(0.73, 1.87)	<0.001	0.17	(−0.29, 0.64)	0.472	−0.07	(−0.18, 0.04)	0.233	−0.01	(−0.10, 0.09)	0.858	
<12 y	423	0.81	(0.59, 1.04)	<0.001	−0.12	(−0.30, 0.07)	0.214	−0.06	(−0.11, −0.02)	0.003	0.03	(−0.003, 0.07)	0.069	
No education	378	0.01	(−0.23, 0.25)	0.923	−0.05	(−0.24, 0.14)	0.579	0.01	(−0.04, 0.05)	0.748	0.02	(−0.01, 0.06)	0.251	
Stratified by primary caregiver														0.132
Caregiver	341	0.76	(0.53, 0.99)	<0.001	−0.01	(−0.21, 0.18)	0.887	−0.05	(−0.10, −0.01)	0.018	0.01	(−0.03, 0.04)	0.731	
Noncaregiver	456	0.33	(0.10, 0.56)	0.005	−0.12	(−0.31, 0.07)	0.227	−0.04	(−0.08, 0.005)	0.081	0.05	(0.01, 0.08)	0.008	
Stratified by cash transfer duration														0.773
2+ y	378	0.30	(0.06, 0.53)	0.013	−0.10	(−0.31, 0.10)	0.330	−0.01	(−0.05, 0.03)	0.599	0.03	(−0.01, 0.06)	0.124	
1 to 2 y	228	0.78	(0.46, 1.10)	<0.001	−0.12	(−0.38, 0.14)	0.352	−0.08	(−0.14, −0.02)	0.007	0.04	(−0.01, 0.09)	0.122	
Less than 1 y	180	0.51	(0.18, 0.85)	0.003	−0.01	(−0.31, 0.28)	0.920	−0.03	(−0.09, 0.03)	0.387	0.01	(−0.04, 0.06)	0.730	
Stratified by CSG eligibility														0.147
Yes	614	0.51	(0.33, 0.70)	<0.001	−0.01	(−0.16, 0.15)	0.918	−0.03	(−0.07, 0.0003)	0.052	0.01	(−0.01, 0.04)	0.297	
No	248	0.41	(0.08, 0.73)	0.014	−0.14	(−0.41, 0.12)	0.299	−0.04	(−0.10, 0.02)	0.245	0.05	(0.01, 0.10)	0.029	
Stratified by OPG eligibility														0.676
Yes	416	0.03	(−0.20, 0.26)	0.797	−0.03	(−0.21, 0.15)	0.74	−0.01	(−0.05, 0.03)	0.649	0.03	(−0.002, 0.07)	0.063	
No	446	0.95	(0.74, 1.16)	<0.001	−0.07	(−0.25, 0.11)	0.456	−0.07	(−0.11, −0.03)	0.001	0.02	(−0.01, 0.06)	0.241	

^*^Model is specified with indicator for Wave 1 to account for practice effects and with mortality/attrition weights.

Subgroup analyses across key sociodemographic categories generally showed positive and stable magnitudes and directions of effect between 0.01 to 0.05 SD units, but were measured imprecisely with CI overlapping the null (Table 2). No statistical differences between subgroups were observed (Wald *P*-values for interaction terms all > 0.1). The cash transfer trended toward having a larger effect in households without children eligible for the CSG (β = 0.05 SD units; 95% CI: 0.01, 0.10) than in households with age-eligible children (β = 0.01 SD unit; 95% CI: −0.03, 0.04; Wald *P*-value for interaction term 0.1; Table 2). The cash transfer effect on memory did not differ by those who were and were not currently eligible for the OPG (β_eligible_ = 0.03 SD units; 95% CI: −0.002, 0.07; β_ineligible_ = 0.02 SD units; 95% CI: −0.01, 0.06; Wald *P*-value for interaction term 0.7; Table 2).The only subgroup without a positive cash transfer slope coefficient was the most educated subgroup (β = −0.01 SD units; 95% CI: −0.1, 0.09), again with CI overlapping the null.

Subgroup analyses by programmatic variables related to the cash transfer trial identified slower memory decline (estimates above the null) for those in the treatment arm across all categories, consistent with the main result (Table 2). The cash transfer trended toward having a larger effect in noncaregivers (β = 0.05 SD units; 95% CI: 0.01, 0.08) than caregivers (β = 0.01 SD unit; 95% CI: −0.03, 0.04; Wald *P*-value for interaction term 0.1; Table 2). The effect was similar in magnitude for those with 1 to 2 y and 2+ y of participation in the cash transfer trial, but smaller for those with less than 1 y of trial participation (Table 2).

### Dementia Probability.

We observed similar results with the dementia probability score outcome (Table 3 and *SI Appendix*, Table S1). In the overall sample at the end of follow-up, dementia probability scores were three percentage points lower in those who received the cash transfer intervention relative to control (β = −0.03 95% CI: −0.05, −0.001). To aid with interpretation of this estimate, we calculated the association between age and dementia probability score at HAALSI Wave 3 in this analytic cohort age 60 y and older. The beta coefficient for age was 0.005 (95% CI: 0.004, 0.006), indicating a 0.5 percentage point increase in dementia probability score for each increasing year of age. Thus, the three percentage point cash transfer effect we observed was roughly equivalent to offsetting the dementia probability increase associated with 6 y of age.

**Table 3.** Impact of the cash transfer intervention on HAALSI Wave 3 dementia probability scores, with coefficients from linear models,^*^ n = 436 older adults age 60 y and older, 2021/22

		Intercept			Cash transfer			
	N	ß	95% CI	*P*-value	ß	95% CI	*P*-value	*P*-value for interaction
Overall	436	0.14	(0.12, 0.16)	<0.001	−0.03	(−0.05, −0.001)	0.045	-
Stratified by sex								0.722
Female	237	0.15	(0.12, 0.17)	<0.001	−0.02	(−0.06, 0.01)	0.254	
Male	199	0.13	(0.10, 0.16)	<0.001	−0.03	(−0.06, 0.01)	0.103	
Stratified by age at HAALSI baseline								0.506
60 to 69 y old	219	0.09	(0.08, 0.10)	<0.001	−0.01	(−0.02, 0.01)	0.339	
70+ y old	218	0.17	(0.14, 0.21)	<0.001	−0.02	(−0.07, 0.02)	0.270	
Stratified by age at HPTN 068 baseline								
<60 y old	222	0.09	(0.08, 0.1)	<0.001	−0.01	(−0.02, 0.01)	0.362	0.500
60+ y old	214	0.18	(0.15, 0.21)	<0.001	−0.02	(−0.07, 0.02)	0.281	
Stratified by household size								0.172
Above median (6)	215	0.12	(0.10, 0.15)	<0.001	−0.01	(−0.04, 0.02)	0.628	
At or below median (6)	221	0.15	(0.13, 0.18)	<0.001	−0.04	(−0.08, −0.003)	0.036	
Stratified by young adult (18 to 25 y) household membership								0.423
Yes	409	0.14	(0.12, 0.16)	<0.001	−0.03	(−0.05, −0.002)	0.033	
No	27	0.11	(0.02, 0.21)	0.025	0.02	(−0.10, 0.13)	0.781	
Stratified by SES								0.434
Above median	233	0.13	(0.11, 0.15)	<0.001	−0.02	(−0.05, 0.01)	0.246	
Below median	203	0.16	(0.13, 0.19)	<0.001	−0.04	(−0.08, 0.01)	0.085	
Stratified by education								0.958
≥12 y	10	0.10	(0.04, 0.16)	0.005	−0.04	(−0.12, 0.04)	0.261	
<12 y	202	0.11	(0.09, 0.14)	<0.001	−0.02	(−0.05, 0.01)	0.148	
No education	223	0.16	(0.13, 0.19)	<0.001	−0.02	(−0.06, 0.02)	0.342	
Stratified by primary caregiver								0.968
Caregiver	166	0.12	(0.10, 0.15)	<0.001	−0.03	(−0.06, 0.01)	0.129	
Noncaregiver	263	0.15	(0.12, 0.17)	<0.001	−0.02	(−0.06, 0.01)	0.164	
Stratified by cash transfer duration								0.931
2+ y	205	0.15	(0.12, 0.18)	<0.001	−0.03	(−0.07, 0.01)	0.209	
1 to 2 y	114	0.13	(0.09, 0.16)	<0.001	−0.02	(−0.06, 0.03)	0.496	
Less than 1 y	102	0.13	(0.09, 0.17)	<0.001	−0.03	(−0.08, 0.03)	0.296	
Stratified by CSG eligibility								0.890
Yes	312	0.14	(0.12, 0.17)	<0.001	−0.03	(−0.06, 0.01)	0.100	
No	124	0.13	(0.10, 0.16)	<0.001	−0.02	(−0.06, 0.02)	0.262	
Stratified by OPG eligibility								0.536
Yes	275	0.16	(0.14, 0.19)	<0.001	−0.03	(−0.06, 0.01)	0.168	
No	161	0.09	(0.07, 0.10)	<0.001	−0.01	(−0.03, 0.01)	0.331	

^*^Model is specified with mortality and attrition weights.

Effect estimates for dementia probability scores were stable across sociodemographic and programmatic subgroups (Table 3). For all sex, age, SES, and education categories, the effect estimates ranged from −0.01 to −0.03. Effect estimates were also in this narrow range for subgroups related to the cash transfer exposure (primary caregiver and cash transfer duration). The Wald *P*-values associated with each subgroup interaction terms were >0.2 (Table 3).

Findings were robust to different assumptions in dementia probability scores among those with missing values (*SI Appendix*, Table S2). The complete case analysis effect estimates were negligibly different from those with missing dementia probability scores imputed at low bounds (β = −0.02; 95% CI: −0.04, 0.003), high bounds (β = −0.04; 95% CI: −0.08, −0.0001), and with multiple imputation (β = −0.03; 95% CI: −0.05, −0.002).

## Discussion

We found that a short-term household cash transfer intervention led to meaningfully slower memory decline and lower dementia probability in a cohort of older adults in a rural South African setting. Over 7 y of follow-up, older adults living in households that received the cash transfer had slower memory decline of a magnitude that would offset 7 y of aging. Cash transfer recipients also had lower dementia probability scores with estimates (11%) similar to those observed in the US Health and Retirement Study (HRS) (10%) (18), suggesting that the intervention brought this older South African population close to the standards observed in a higher-resourced setting. These effects were largely consistent in magnitude and direction across sociodemographic groups, suggesting that the intervention efficacy did not differ by age, sex, baseline SES, or educational attainment. Key strengths of this study are its randomized cash transfer intervention and evaluation of long-term and rigorously measured ADRD outcomes.

Our findings align with the positive relationships observed between cash transfers and cognitive health in five prior studies worldwide. A variety of natural experiment and observational approaches have revealed a positive relationship between pension receipt and cross-sectional cognitive function in the United States and South Africa (13, 14), and longitudinal cognitive and memory function in South Korea and China (15, 17). Cluster-randomized evidence from Mexico also revealed links between pension exposure and slower memory decline, but was limited to two waves of data collection with <1 y of follow-up (16). Our findings extend the reach of this prior work with 7 y of longitudinal follow-up across three waves, with rigorous memory decline and dementia probability outcomes, and with household-level randomization of the cash transfer exposure.

Our observation of slightly larger memory effects in noncaregivers relative to caregivers was unexpected. This difference was not statistically significant, but the magnitude of it warrants comment. Primary caregivers to the HPTN 068 index young women would have been the direct recipients of 2/3 of the monthly cash transfer amount. We thus expected that the caregivers would benefit more than noncaregivers who would receive benefits through indirect household spillover mechanisms only. We speculate that these differences may be due to the age and sex composition of caregivers vs. noncaregivers. Caregivers were most likely to be mothers of index young women, which placed them largely in the female and younger age categories. Both of these groups trended toward smaller cash transfer effects, potentially explained by the observed lower risk of memory decline at younger ages, and findings that women tend to invest household money in other household members instead of themselves (19). It also tracks with the larger effects observed in households without children eligible for the CSG. Future work should seek to disentangle the effects of age, gender, and household role from the direct vs. indirect cash transfer pathways.

The effect of the cash transfer intervention on cognitive outcomes was similar for those who were and were not eligible to receive the OPG at the time of the intervention and at the time of cognitive follow-up. These results are counter to the hypothesis that receiving cash transfer income from two sources would result in better outcomes than receiving cash transfer income from a single source. The randomized cash transfer intervention, with a R300 monthly payment, was also substantially less generous than monthly payments from the OPG, which increased from R1140 in 2011 to R2090 in 2023. We outline two plausible explanations for this unexpected finding. First, the life course timing of cash transfer exposures may be an important determinant of their impact on later life cognitive functioning. South African adults are eligible for the OPG at age 60 y and older while the randomized cash transfer was delivered in earlier adulthood for those who received it in isolation from the OPG. Our findings that this smaller cash transfer payment delivered in mid-adulthood produced impacts comparable to those observed with larger combined payments in later-adulthood are consistent with a sensitive life course period in mid-adulthood. Mid-adulthood may coincide with the stressors of raising children and, in this study population, of living in a high unemployment setting at an age prior to eligibility for the OPG as a social safety net. Both of these stressors could plausibly contribute to a depletion of cognitive reserve (7), and could be responsive to a cash transfer intervention. Our findings align with a Rush Memory and Aging study that found higher socioeconomic status at all adult life course periods was positively related to later-life cognitive function level (20). Second, the OPG exposure was not randomized and is structurally related to older age due to the age eligibility requirement. The rate of memory decline and dementia probability can accelerate at older ages (e.g., beyond age ~65) (21, 22). Thus, the older group eligible to receive the OPG may not have experienced a stronger effect of receiving an additional cash transfer as they faced stronger cognitive aging pressures than their younger counterparts. Future work should seek to better understand the incremental impacts of cash transfer income when accumulated over multiple sources.

A priority area for future work will be to understand the mechanisms through which cash transfers operate to produce cognitive health impacts. Our finding of a significant total effect of the randomized cash transfer is compatible with our hypothesized pathway through investments in cognitively stimulating exposures that build up cognitive reserve (7). This pathway should be confirmed in future work by operationalizing cognitive reserve and empirically assessing how it is related to cash transfer exposures (23). Cash transfer policy discussions and programming decisions would also benefit from understanding which investments made by cash transfer recipients best protect their cognitive health. As such, mediation pathways should be investigated to contextualize the relative strength of different spending patterns and asset accumulation in ADRD prevention (e.g., through spending on leisure activities, items that promote social connections, healthcare utilization, and nutritious food) (8, 9).

Our results should be interpreted in the context of the specific study population and structure of the cash transfer intervention under study. The study population was older adults from a rural community in South Africa. Unique life course experiences that shape the cognitive health of this cohort include the HIV epidemic and life course educational and economic deprivation stemming from Apartheid (24, 25). This study community also had access to a family of government-supported cash transfer programs (26, 27) in addition to the experimental cash transfer under study. To be included in our analytic cohort, older adults had to live in households with a trial-eligible and -enrolled young woman. This restriction will not be representative of the household composition of all older adults. All these factors could influence the strength of effect and pathways through which the cash transfer influences ADRD outcomes. Thus, the generalizability of our findings should be confirmed in other populations and with other cash transfer intervention designs.

Finally, household members had to survive to enroll in HAALSI to be included in our analytic sample. Prebaseline deaths could be influential to the inference we draw from our findings if they were systematically different from those who survived to enroll in HAALSI. The end of the HPTN 068 cash transfer trial coincided with Wave 1 of HAALSI in 2015, which minimizes the opportunity for prebaseline deaths to have accumulated in the interim. Nonetheless, given the strong relationship between poor cognitive health and mortality, the potential for prebaseline survival bias is important to consider in interpreting our findings.

## Conclusion

LMICs are projected to account for the largest share of global ADRD cases and associated economic burden by 2050 (2, 4). In this low-income setting in rural South Africa, we found that a modestly sized cash transfer delivered over a short period in mid- to later-life led to a meaningful slowing of memory decline and reduction in dementia probability 7 y later. Investments in new and expanded cash transfer programs could help to stem the tide of new ADRD cases in LMIC settings. Questions remain about the optimal life course timing, duration, and amount of cash transfers to maximize population-level ADRD prevention outcomes. It may be that targeting to different life course periods, longer durations, or larger monthly payouts would produce stronger results. Although ADRD prevention is one of many health and well-being outcomes that may be a consideration for cash transfer policymakers, the extreme projections for ADRD in LMIC settings make these questions particularly salient and a priority area for future ADRD research.

## Methods

### Study Setting.

The study population is from a rural community in Mpumalanga province in northeastern South Africa, an area of the country where Black South Africans were forcibly relocated under the Apartheid period (1948 to 1994) of legislated racial segregation. The older adult population living in the area have lived lives with limited and low-quality educational opportunities and high unemployment. Temporary and permanent migration out of the community for employment opportunities is widespread among men and increasingly women, with associated remittances important sources of household income. Other important income sources are governmental social grants. South Africa has a rich suite of social grants, the largest of which are the CSG (CSG, designed to alleviate poverty by offsetting the costs of raising children, age eligibility: 0 to 18 y, monthly payment: R510/~$27 USD) (27) and the OPG (designed to alleviate poverty among the aging population, age eligibility: 60+ y, monthly payment: R2090/~$110 USD) (28). Eligibility for both of these programs is restricted to those who meet a means test which is almost universally met by the households in this low-income area. In this setting, the Agincourt Health and socio-Demographic Surveillance System (HDSS) has been operating since 1992 (25). The Agincourt HDSS conducts continuous surveillance of vital events, sociodemographics, health outcomes, and behaviors in the full population of ~117,000 people living across 31 study villages. The Agincourt HDSS is the common sampling frame that allows for the linkage of the two studies we leveraged in this paper: HPTN 068 and HAALSI.

### HPTN 068.

The HPTN study 068 was a Phase 3 randomized controlled trial to examine the impact of a conditional cash transfer intervention on HIV incidence in young women (29, 30). In 2011, n = 2,533 young women enrolled in the trial with their primary caregivers. Key eligibility criteria for the young women were age 13 to 20 y, enrolled in school (grade 8 to 11), and not married or pregnant. They were also required to live with their primary caregiver and both be able to open a bank or post office account. Young women and their primary caregivers were randomly assigned 1:1 to the cash transfer intervention or control arm using block randomization. Due to the nature of the intervention, participants and their households were not masked to their assignment.

The cash transfer intervention was a monthly cash payment of R300 [~36 USD at time of study, anchored to the amount of the governmental CSG monthly payments (27)], conditional on >80% school attendance in the prior month. In practice, nearly all young women in the trial met this condition (95.1 and 95.5% in intervention and control arms). Thus, even though the cash transfer was conditioned on school attendance, in practice, the intervention did not appear to influence attendance above the high background rates, and the payments were close to universal (29). Importantly, the payments were split between the young woman and her primary caregiver with the young woman receiving 1/3 of each month’s payment directly and 2/3 of it paid to the caregiver. The R300 monthly payment represented a meaningful increase in income for trial households. Baseline monthly per capita consumption in intervention households was R455, about half of which (R235) was in food consumption, almost R100 lower than the 2011 South African food poverty line (31). The R300 monthly payment is thus of a magnitude that could have moved multiple household members toward the food consumption thresholds necessary to meet basic needs.

### HAALSI Cohort.

HAALSI is a population-representative longitudinal cohort study broadly designed to characterize health and aging in this rural Agincourt setting (32, 33). Between November 2014 and November 2015, a total of n = 5,059 men and women enrolled in HAALSI, drawn from a random sample of all adults aged 40 y and older who permanently lived in the study site during the previous 12 mo. HAALSI study procedures included in-home surveys with participants, with a cognitive battery harmonized with that of the US HRS. Surveys were administered by local, trained fieldworkers in the local Xitsonga language. A second and third HAALSI wave were conducted in 2018/19 and 2021/22, respectively, for a total of 7 y of follow-up to date.

### Analytic Cohort Construction.

Using the shared Agincourt HDSS sampling frame for HPTN 068 and HAALSI, we identified study households involved in the cash transfer trial that included older adult members who went on to enroll in HAALSI (n = 862 individuals across n = 724 unique households).

### Ethical Approvals.

The protocols for HPTN 068 and HAALSI were reviewed and approved by Institutional Review Boards at the and University of the Witwatersrand and the Mpumalanga Provincial Research and Ethics Committee. The protocols were additionally reviewed by the University of North Carolina-Chapel Hill (HPTN 068) and the Harvard T.H. Chan School of Public Health (HAALSI). The protocol for this secondary analysis was reviewed and approved by the Institutional Review Board at Indiana University (deemed “not human subjects research” with its use of fully deidentified datasets: #2002584956), and the University of the Witwatersrand (#M20056).

### Key Measures.

Our exposure of interest was the randomly assigned intervention arm for HPTN 068. We extracted the allocation from the HPTN 068 dataset and merged it with household information maintained in the Agincourt HDSS census records. All older adult HAALSI members who lived in HPTN 068 trial households were assigned the intervention vs. control arm status of their household. Those living in the intervention households received the cash transfer intervention while those living in control households did not. Individuals in our intervention households could have received income from the cash transfer directly if they were the primary caregiver of the index young woman, or indirectly through household spillover mechanisms (e.g., received food or other resources purchased with the income or had more disposable income because no longer responsible for costs like school uniforms or textbooks paid for with the transfer).

We used two primary outcomes of interest:

1) Memory decline: We examined rate of memory change over time (“decline”) because loss of memory function is one of the earliest and hallmark symptoms of ADRD (34). The HAALSI survey assessed memory function at each of the three waves in 2014/15, 2018/19, and 2021/22. In all waves, episodic memory was assessed with immediate and delayed word recall trial(s) of a 10-word list read aloud by the interviewer, with a protocol adapted from the US HRS and implemented in International Partner Studies of the HRS around the world (35, 36). The distribution observed in HAALSI is similar to distributions observed in HRS partner studies in Mexico, India, and China, all of which had distributions shifted to the left of those observed in the United States and England, indicating lower population cognitive performance (37). In Wave 1, interviewers conducted a single immediate word recall trial followed by a delayed word recall trial ~2 min later. In Waves 2 to 3, interviewers conducted three immediate recall trials with a delayed recall trial 13 min later. As the number and administration of the trials differed between waves, we used confirmatory factor analysis to derive a single factor to represent episodic memory across all three waves drawing on all available recall trial data at each wave (38). We constrained the factor loadings on items comparable across waves and allowed the model to impute missing data. Wave-specific scores were thus calculated for all HAALSI participants with nonproxy interviews and with data for at least one recall trial at that wave. The model was a good fit for the data (RMSEA: 0.051, CFI: 0.978, TLI: 0.970, SRMR: 0.03). We z-standardized the episodic memory factor score at Wave 1 (mean = 0, SD = 1) and then standardized the scores at Waves 2 and 3 to the distribution at Wave 1. We defined memory decline as the memory score slope from Wave 1 to Wave 3, modeled as described in the statistical analysis section below.
2) Dementia probability score: Consensus-based dementia diagnoses from the HAALSI Dementia subcohort (n = 635) were used as a “gold standard” to generate dementia probability scores for the entire HAALSI cohort (39, 40). A multidisciplinary expert panel was assembled to assign dementia diagnoses among subcohort participants. The panel included US and South African neuropsychiatrists, geriatricians, and neurologists. We developed and optimized a predictive model within the HAALSI Dementia subcohort, drawing on measures in the Wave 2 survey to predict subsequent dementia diagnosis. The best-performing model (area under the receiver operating characteristic curve = 0.79) included a battery of cognitive test scores (immediate and delayed recall, orientation, verbal fluency, sum score of days of the week forward and backward), self-rated memory activities of daily living, and instrumental activities of daily living. We used this model to predict the probability of dementia (theoretical range: 0 to 100%) in the full HAALSI sample at Wave 3, drawing on their observed demographic, cognitive, and behavioral data.
As the dementia probability score calculation depended on complete data for covariates in the predictive model, scores were missing for those with missing covariates (n = 106, 16% of the sample with follow-up through Wave 3). We explored the sensitivity of our findings to these missing data by imputing dementia probability scores under different assumptions about the unobserved dementia probabilities. We imputed missing values assuming all had dementia probability in the lowest quartile (randomly selected between 1.1 to 4.6%, “low bounds”) and highest quartile (randomly selected between 11.5 to 88.5%, “high bounds”), and by using multiple imputation, drawing on the information in observed covariates to predict missing dementia probability scores. Thirty datasets were imputed using Fully Conditional Specification Multiple Imputation, drawing on the predictive power of key covariates (age, sex, household wealth, education, marital status, and literacy) (41).

We used a set of covariates to contextualize the study population and test whether the effect of the cash transfer intervention differed in certain subgroups. We examined the following covariates recorded in the HAALSI baseline survey: age (in years, categorized at HAALSI baseline at <50, 50 to 69, and 70+ for memory decline and at <70 vs. 70+ for dementia probability, and at HPTN 068 baseline at <60 and 60+ for both outcomes to align with age-eligibility for the OPG at the start of the intervention), sex (male vs. female), nationality of birth (South Africa vs. all others), marital status (divorced/separated, married/cohabitating, single, or widowed), household wealth (measured with a wealth index created from principle components analysis of household assets then categorized in quintiles), educational attainment (no education, <12 y and ≥ 12 y), whether household had a young adult (age 18 to 25) in residence, household eligibility for the CSG (based on households with age-eligible children at HAALSI baseline), and individual eligibility for the OPG (based on the participant being age 60+ y at HAALSI baseline).

We also examined variables that we defined by linking the HAALSI participant to Agincourt HDSS census records and the HPTN 068 dataset. We examined the relationship of the HAALSI participant to the index young woman (categorized as father, mother, grandfather, grandmother, or other). Because a portion of the cash transfer was paid directly to the primary caregiver of the index young woman, we further examined whether our participants were recorded as the primary caregiver (yes vs. no). However, the primary caregiver status was only recorded in census records for parents or grandparents. Household members with “other” relationships with the index young women were recorded as nonprimary caregivers if a primary caregiver was otherwise identified, or missing if not. Finally, we examined the amount of time each household received the cash transfer (categorized as <1 y, 1 to 2 y, and 2+ y). Index trial participants became ineligible after finishing 12th grade, so young women who enrolled in later grades had less time in the trial than those who enrolled in earlier grades. To create an appropriate comparison in control households, we assigned durations of <1 y to households with young women in 11th grade, 1 to 2 y in 10th grade, and 2 + y in 8 to 9th grade.

### Statistical Analysis.

To estimate the impact of the cash transfer intervention on memory decline, we constructed linear mixed models with subject-specific random intercepts, household-specific random intercepts to account for multiple participants in the same household, and random slopes for time to model the change in memory score between Wave 1 and Wave 3. The dependent variable was memory score, predicted by randomized cash transfer intervention arm, exact time since baseline interview in years, and the interaction between cash transfer intervention arm and time. We interpret this interaction term coefficient as the difference in memory slope for the intervention vs. control arm. To account for practice effects in memory scores observed after the baseline assessment, we incorporated an indicator variable for HAALSI Wave 1 observations (42). To explore nonlinear trends in the cash transfer/memory function relationship over time, we specified an alternative model with time coded in discrete indicator variables for wave and calculated the difference in mean memory score at each wave. Although this specification allowed us to more flexibly model the relationship over time, it does not correct for practice effects, so results are not directly comparable to the main model.

To estimate the impact of the cash transfer intervention on dementia probability, we constructed linear models with robust SE to account for multiple participants in the same household specified to predict dementia probability at HAALSI Wave 3 from the randomized cash transfer intervention arm. We interpret the coefficient associated with the intervention arm as the difference in dementia probability for the intervention vs. control arm. As dementia probability was low among those younger than 60 y (mean 6.7%), we restricted the analytic cohort for the dementia probability analysis to those age 60 and above at Wave 3 (n = 436).

We stratified models for both outcomes by key sociodemographic and programmatic covariates: sex, age (in categories <50, 50 to 69, and 70+ y), SES (categorized above/below median household asset index), educational attainment, primary caregiver status, duration of cash transfer receipt, household size (above/below median), young adult in household (yes/no), household eligibility for the CSG, and individual eligibility for the OPG. We did not incorporate additional covariate adjustment into the models based on the randomized nature of the exposure. To minimize the potential for bias from differential mortality and attrition, we applied inverse probability of censoring weights to all models, combining weights separately constructed for mortality and study drop-out processes between Wave 1 and Wave 3 (42).

## Supplementary Material

Appendix 01 (PDF)

## Data Availability

Some study data are available. The HAALSI data analyzed in this paper are publicly accessible at https://haalsi.org/data (33). The HPTN 068 data analyzed in this paper are publicly accessible at: https://dataverse.harvard.edu/dataverse/HPTN068 (30). The linkage between the HAALSI and HPTN 068 cohorts is maintained by the Agincourt Health and Demographic Surveillance System (AHDSS). AHDSS data are available upon request as described on their data overview website (http://www.agincourt.co.za/index.php/data/) (43) and by contacting their data manager at DataManager@agincourt.co.za.

## References

[1] E. Nichols , Estimation of the global prevalence of dementia in 2019 and forecasted prevalence in 2050: An analysis for the Global Burden of Disease Study 2019. Lancet Public Health **7**, e105–e125 (2022). 34998485 10.1016/S2468-2667(21)00249-8 PMC8810394

[2] C. Patterson, “World Alzheimer report 2018” (Alzheimer's Disease International, London, UK, 2018).

[3] D. Bloom , “Addressing Alzheimer’s disease and related dementias to realise the promise of the UN’s ‘Decade of Healthy Ageing’” (VoxEU.org, London, UK, 2021), **vol. 26**.

[4] A. Nandi , Global and regional projections of the economic burden of Alzheimer’s disease and related dementias from 2019 to 2050: A value of statistical life approach. EClinicalMedicine **51**, 101580 (2022). 35898316 10.1016/j.eclinm.2022.101580 PMC9310134

[5] J. Mah, K. Rockwood, S. Stevens, J. Keefe, M. K. Andrew, Do interventions reducing social vulnerability improve health in community dwelling older adults? A systematic review. Clin. Interv. Aging **17**, 447–465 (2022). 35431543 10.2147/CIA.S349836 PMC9012306

[6] J. R. Marden, E. J. Tchetgen Tchetgen, I. Kawachi, M. M. Glymour, Contribution of socioeconomic status at 3 life-course periods to late-life memory function and decline: Early and late predictors of dementia risk. Am. J. Epidemiol. **186**, 805–814 (2017). 28541410 10.1093/aje/kwx155 PMC5859987

[7] Y. Stern, What is cognitive reserve? Theory and research application of the reserve concept. J. Int. Neuropsychol. Soc. **8**, 448–460 (2002). 11939702

[8] L. C. Kobayashi , Long-term household material socioeconomic resources and cognitive health in a population-based cohort of older adults in rural northeast South Africa, 2001–2015. SSM Popul. Health **20**, 101263 (2022). 36281246 10.1016/j.ssmph.2022.101263 PMC9587313

[9] A. Jennings, S. C. Cunnane, A. M. Minihane, Can nutrition support healthy cognitive ageing and reduce dementia risk? BMJ **369**, m2269 (2020). 32591407 10.1136/bmj.m2269 PMC7318880

[10] F. Bastagli , “Cash transfers: What does the evidence say. A rigorous review of programme impact and the role of design and implementation features” (ODI, London, 2016), **vol. 1**.

[11] C. R. Herl, C. Kabudula, K. Kahn, S. Tollman, D. Canning, Pension exposure and health: Evidence from a longitudinal study in South Africa. J. Econ. Ageing **23**, 100411 (2022). 10.1016/j.jeoa.2022.100411 PMC9731801 36505964

[12] M. Rosenberg , The impact of a randomized cash transfer intervention on mortality of adult household members in rural South Africa, 2011–2022. Soc. Sci. Med. **324**, 115883 (2023). 37023659 10.1016/j.socscimed.2023.115883 PMC10124166

[13] P. Ayyagari, D. Frisvold, The impact of social security income on cognitive function at older ages. Am. J. Health Econ. **2**, 463–488 (2016).

[14] J. Jock , Effects of pension eligibility expansion on men’s cognitive function: Findings from rural South Africa. J. Aging Soc. Policy 1–20 (2023), https://doi.org/10.1080/08959420.2023.2195785. 10.1080/08959420.2023.2195785 PMC10533724 36975023

[15] I. Hwang, T.-J. Lee, Health improvements of older adults based on benefit duration: Lessons from Korean social pension policies. Soc. Sci. Med. **315**, 115514 (2022). 36395599 10.1016/j.socscimed.2022.115514

[16] E. Aguila, M. Casanova, Short-term impact of income on cognitive function: Evidence from a sample of Mexican older adults. J. Aging Health **32**, 591–603 (2020). 30947596 10.1177/0898264319841155

[17] C. Peng, J. A. Burr, S. H. Han, Cognitive function and cognitive decline among older rural Chinese adults: The roles of social support, pension benefits, and medical insurance. Aging Ment. Health **27**, 771–779 (2023). 35702759 10.1080/13607863.2022.2088693 PMC10460523

[18] J. J. Manly , Estimating the prevalence of dementia and mild cognitive impairment in the US: The 2016 health and retirement study harmonized cognitive assessment protocol project. JAMA Neurol. **79**, 1242–1249 (2022). 36279130 10.1001/jamaneurol.2022.3543 PMC9593315

[19] E. Duflo, Grandmothers and granddaughters: Old-age pensions and intrahousehold allocation in South Africa. World Bank Econ. Rev. **17**, 1–25 (2003).

[20] A. Krasnova, S. E. Tom, L. Valeri, P. K. Crane, D. A. Bennett, Direct effect of life-course socioeconomic status on late-life cognition and cognitive decline in the Rush Memory and Aging Project. Am. J. Epidemiol. **192**, 882–894 (2023). 36757185 10.1093/aje/kwad033 PMC10505419

[21] T. A. Salthouse, Trajectories of normal cognitive aging. Psychol. Aging **34**, 17 (2019). 30211596 10.1037/pag0000288 PMC6367038

[22] R. S. Wilson, T. Wang, L. Yu, D. A. Bennett, P. A. Boyle, Normative cognitive decline in old age. Ann. Neurol. **87**, 816–829 (2020). 32144793 10.1002/ana.25711 PMC10035056

[23] B. R. Reed , Measuring cognitive reserve based on the decomposition of episodic memory variance. Brain **133**, 2196–2209 (2010). 20591858 10.1093/brain/awq154 PMC3139935

[24] M. S. Rosenberg , Sexual behaviors and HIV status: A population-based study among older adults in rural South Africa. J. Acquir. Immune Defic. Syndr. **74**, e9 (2017). 27926667 10.1097/QAI.0000000000001173 PMC5147032

[25] K. Kahn , Profile: Agincourt health and socio-demographic surveillance system. Int. J. Epidemiol. **41**, 988–1001 (2012). 22933647 10.1093/ije/dys115 PMC3429877

[26] A. Case, A. Deaton, Large cash transfers to the elderly in South Africa. Econ. J. **108**, 1330–1361 (1998).

[27] J. D. Triegaardt, The Child Support Grant in South Africa: A social policy for poverty alleviation? Int. J. Soc. Welf. **14**, 249–255 (2005).

[28] M. Ralston, E. Schatz, J. Menken, F. X. Gómez-Olivé, S. Tollman, Who benefits—or does not—from South Africa’s old age pension? Evidence from characteristics of rural pensioners and non-pensioners. Int. J. Environ. Res. Public Health **13**, 85 (2016). 10.3390/ijerph13010085 PMC4730476 26712777

[29] A. Pettifor , The effect of a conditional cash transfer on HIV incidence in young women in rural South Africa (HPTN 068): A phase 3, randomised controlled trial. Lancet Glob. Health **4**, e978–e988 (2016). 27815148 10.1016/S2214-109X(16)30253-4 PMC5626439

[30] L. Emel, “Main Study HPTN 068 computer-assisted self-interview survey data”. Harvard Dataverse, V1. 10.7910/DVN/YEOHWG. Deposited 17 July 2024.

[31] K. Kilburn , Cash transfers, young women’s economic well-being, and HIV risk: Evidence from HPTN 068. AIDS Behav. **23**, 1178–1194 (2019). 30415429 10.1007/s10461-018-2329-5 PMC6510655

[32] F. X. Gómez-Olivé , Cohort profile: Health and ageing in Africa: A longitudinal study of an indepth community in South Africa (HAALSI). Int. J. Epidemiol. **47**, 689–690j (2018). 29325152 10.1093/ije/dyx247 PMC6005147

[33] Harvard Center for Population and Development Studies (Harvard T.H. Chan School of Public Health), HAALSI Wave 3 Survey. Harvard Dataverse, V2. 10.7910/DVN/Q2JFPV. Deposited 19 May 2023.

[34] J. Bowen , Progression to dementia in patients with isolated memory loss. Lancet **349**, 763–765 (1997). 9074575 10.1016/S0140-6736(96)08256-6

[35] M. B. Ofstedal, G. G. Fisher, A. R. Herzog, “Documentation of cognitive functioning measures in the Health and Retirement Study” (Tech. Rep. DR-006, University of Michigan, Ann Arbor, MI, 2005), 10, 1114577250.1662476251-1381895763.1662476251.

[36] L. C. Kobayashi , You say tomato, I say radish: Can brief cognitive assessments in the US health retirement study be harmonized with its international partner studies? J. Gerontol. B Psychol. Sci. Soc. Sci. **76**, 1767–1776 (2021). 33249448 10.1093/geronb/gbaa205 PMC8557836

[37] A. L. Gross , Harmonisation of later-life cognitive function across national contexts: Results from the Harmonized Cognitive Assessment Protocols. Lancet Healthy Longev. **4**, e573–e583 (2023). 37804847 10.1016/S2666-7568(23)00170-8 PMC10637129

[38] A. L. Gross , Application of latent variable methods to the study of cognitive decline when tests change over time. Epidemiology **26**, 878 (2015). 26414855 10.1097/EDE.0000000000000379 PMC4819068

[39] D. T. Bassil , Cohort Profile Update: Cognition and dementia in the Health and Aging in Africa Longitudinal Study of an INDEPTH community in South Africa (HAALSI dementia). Int. J. Epidemiol. **51**, e217–e226 (2022). 34871405 10.1093/ije/dyab250 PMC9365629

[40] M. T. Farrell , Estimating dementia prevalence using remote diagnoses and algorithmic modeling: A population-based study of a rural region in South Africa. Lancet Glob. Health, in press. 10.1016/S2214-109X(24)00325-5 PMC11987161 39577957

[41] S. Van Buuren, Multiple imputation of discrete and continuous data by fully conditional specification. Stat. Methods Med. Res. **16**, 219–242 (2007). 17621469 10.1177/0962280206074463

[42] J. Weuve , Guidelines for reporting methodological challenges and evaluating potential bias in dementia research. Alzheimers Dement. **11**, 1098–1109 (2015). 26397878 10.1016/j.jalz.2015.06.1885 PMC4655106

[43] MRC/Wits-Agincourt, Data Overview: For researchers wishing to analyse data collected at the Agincourt HDSS there are a number of mechanisms in place to facilitate access to the data. http://www.agincourt.co.za/index.php/data/. Accessed 22 August 2024.

