# Supplementary material for: Effect of a cash transfer intervention on memory decline and dementia probability in older adults in rural South Africa
Source: Proc Natl Acad Sci U S A. 2024 Sep 19;121(40):e2321078121. doi: 10.1073/pnas.2321078121 (PMC11459187; doi:10.1073/pnas.2321078121)
Supplement: Supplementary file 1 — Appendix 01 (PDF) [file pnas.2321078121.sapp.pdf]

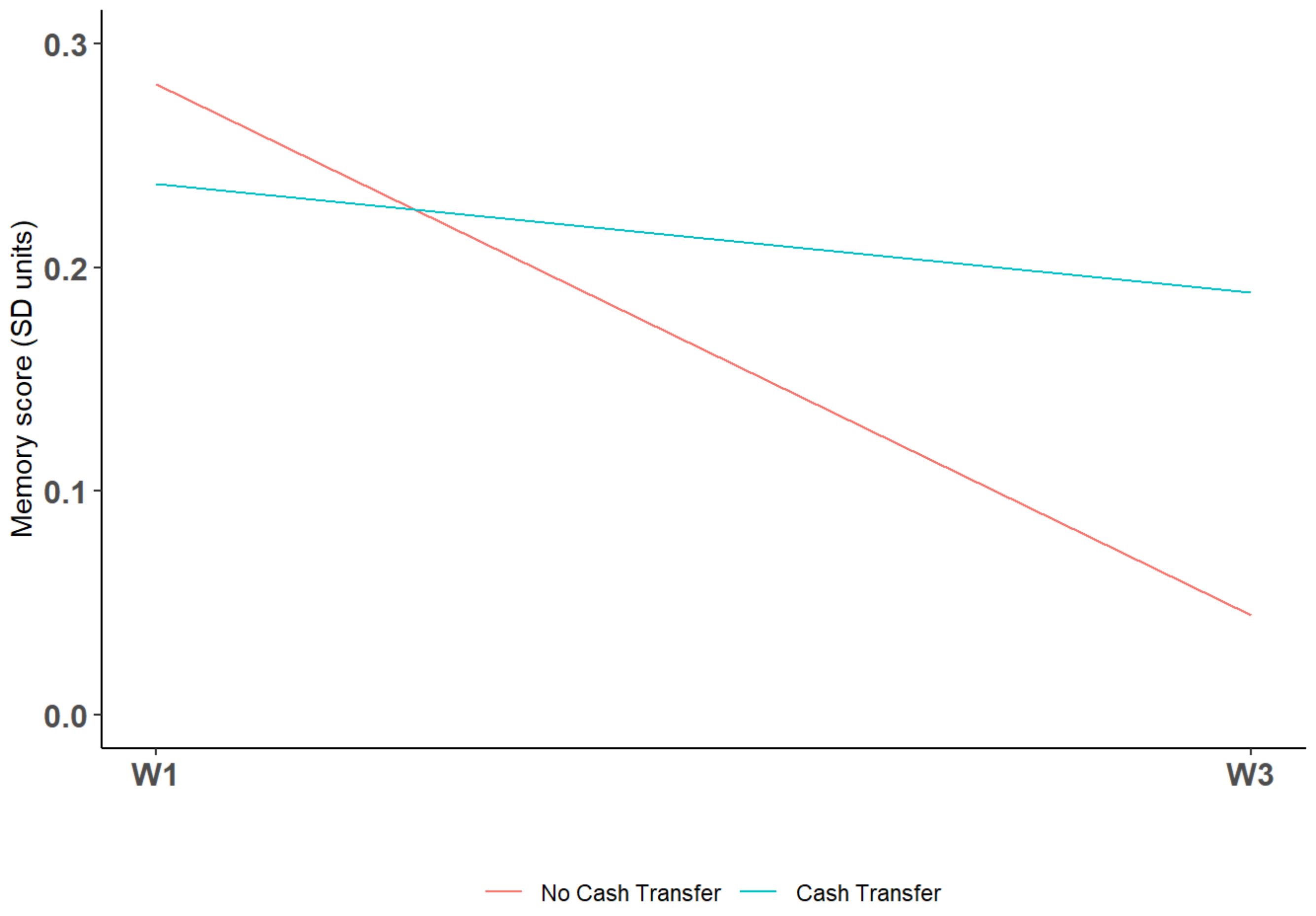

Figure S1. Graphical representation of impact of the cash transfer intervention on memory slopes, drawn from a generalized linear mixed model[1], n=862 older adults age 40 years and older, 2014-2022

[1]Model is specified with indicator for Wave 1 to account for practice effects and with mortality/attrition weights

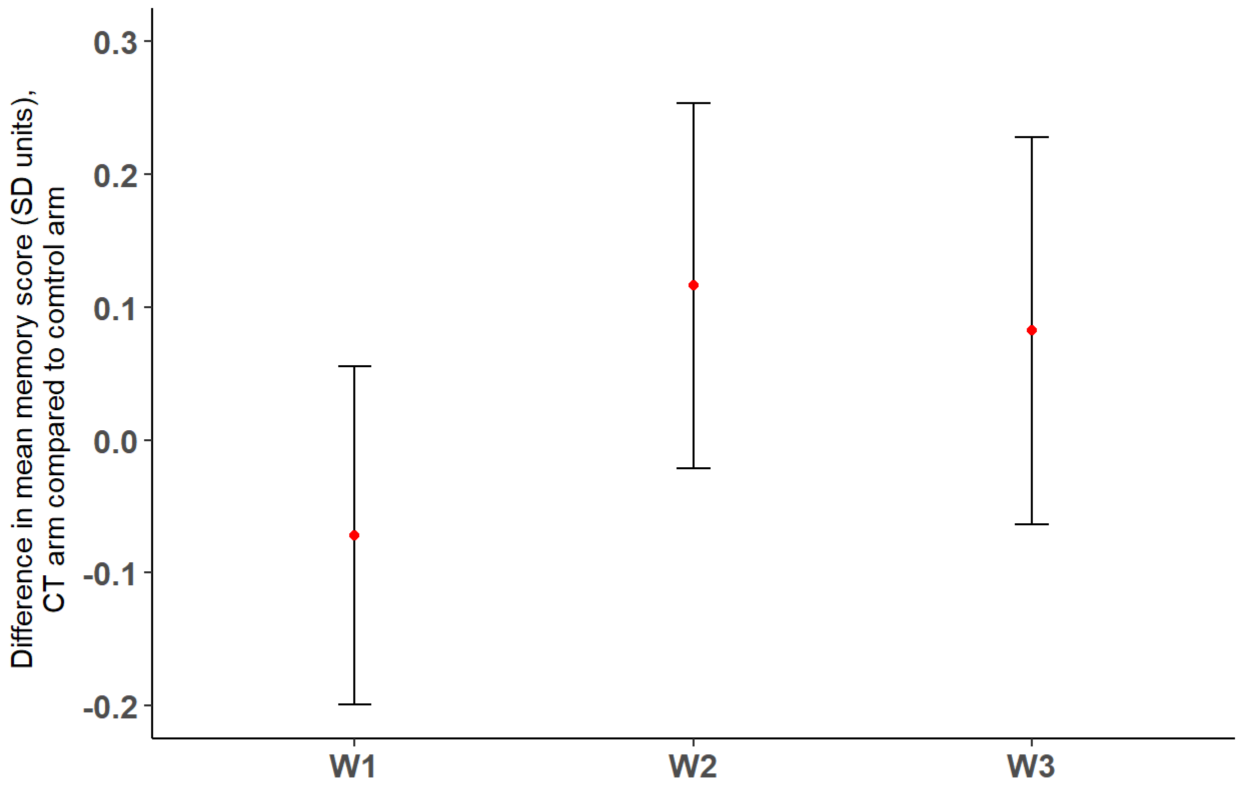

Figure S2. Predicted difference in mean memory scores across Waves 1-3[1], by cash transfer intervention arm

[1]From a LMM with mortality/attrition weights, dummy variables for wave, interaction between each wave and cash transfer (CT) trial arm, subject-specific random intercepts, and household-specific random intercepts. This model does not adjust for practice effects.

Table S1. Predicted mean memory scores across Waves 1-3[1] and predicted dementia probability at Wave 3[2], by cash transfer intervention arm

	**Total (n = 862)**	**Cash Transfer (n = 429)**	**No Cash Transfer (n = 433)**
	Predicted mean (95% CI)	*Predicted mean (95% CI)*	*Predicted mean (95% CI)*
**Memory score** (W1)	0.04 (-0.02, 0.11)	0.01 (-0.08, 0.10)	0.08 (-0.01, 0.17)
**Memory score** (W2)	0.39 (0.32, 0.46)	0.45 (0.35, 0.54)	0.33 (0.23, 0.43)
**Memory score** (W3)	0.33 (0.26, 0.40)	0.37 (0.27, 0.48)	0.29 (0.19, 0.39)
**Dementia probability** (W3)[2]	0.13 (0.11, 0.14)	0.11 (0.10, 0.13)	0.14 (0.12, 0.16)

[1]From a linear mixed model, specified with mortality/attrition weights, discrete indicator variables for wave, interaction between each wave and CT arm, subject-specific random intercepts, and household-specific random intercepts. This model does not adjust for practice effects.

[2]From a linear model with mortality/attrition weights and with standard errors clustered at the household-level

Table S2. Robustness check to explore the sensitivity of model output[1] to assumptions about missing HAALSI Wave 3 dementia probability scores

	Intercept			Cash transfer		
	**ß**	**95% CI**	**p-value**	**ß**	**95% CI**	**p-value**
**Complete case**	0.14	(0.12, 0.16)	<0.001	-0.03	(-0.05, -0.001)	0.045
**Low bound[2]**	0.13	(0.11, 0.14)	<0.001	-0.02	(-0.04, 0.003)	0.096
**High bound[3]**	0.20	(0.18, 0.23)	<0.001	-0.04	(-0.08, -0.0001)	0.049
**Multiple imputation[4]**	0.15	(0.13, 0.16)	<0.001	-0.03	(-0.05, -0.002)	0.031

[1]Model is specified with mortality and attrition weights
[2]Observations with missing dementia probability scores assigned scores in the lowest quartile (randomly selected between 1.2-5.4%)
[3]Observations with missing dementia probability scores assigned scores in the highest quartile (randomly selected between 13.8-88.5%)
[4]Missing dementia probability scores imputed using multiple imputation
